# AUDIOVISUAL RESOURCES ON THE TEACHING PROCESS IN SURGICAL TECHNIQUE

**DOI:** 10.1590/S0102-6720201500040004

**Published:** 2015

**Authors:** Guilherme Luiz Lenzi PUPULIM, Rafael Augusto IORIS, Ricardo Ribeiro GAMA, Carmen Australia Paredes Marcondes RIBAS, Osvaldo MALAFAIA, Mirnaluci GAMA

**Affiliations:** Evangelic Faculty of Paraná Medical School and Postgraduate Program in Principles of Surgery, Curitiba, PR, Brazil

**Keywords:** Medicine, Teaching, Education, Medical, Undergraduate, Audiovisual Aids, Surgical Procedures,Operative

## Abstract

**Background:**

The development of didactic means to create opportunities to permit complete and repetitive viewing of surgical procedures is of great importance nowadays due to the increasing difficulty of doing in vivo training. Thus, audiovisual resources favor the maximization of living resources used in education, and minimize problems arising only with verbalism.

**Aim:**

To evaluate the use of digital video as a pedagogical strategy in surgical technique teaching in medical education.

**Methods:**

Cross-sectional study with 48 students of the third year of medicine, when studying in the surgical technique discipline. They were divided into two groups with 12 in pairs, both subject to the conventional method of teaching, and one of them also exposed to alternative method (video) showing the technical details. All students did phlebotomy in the experimental laboratory, with evaluation and assistance of the teacher/monitor while running. Finally, they answered a self-administered questionnaire related to teaching method when performing the operation.

**Results:**

Most of those who did not watch the video took longer time to execute the procedure, did more questions and needed more faculty assistance. The total exposed to video followed the chronology of implementation and approved the new method; 95.83% felt able to repeat the procedure by themselves, and 62.5% of those students that only had the conventional method reported having regular capacity of technique assimilation. In both groups mentioned having regular difficulty, but those who have not seen the video had more difficulty in performing the technique.

**Conclusion:**

The traditional method of teaching associated with the video favored the ability to understand and transmitted safety, particularly because it is activity that requires technical skill. The technique with video visualization motivated and arouse interest, facilitated the understanding and memorization of the steps for procedure implementation, benefiting the students performance.

## Abstract (pt)

**Racional:**

O desenvolvimento de meios que permitam a completa e repetitiva visibilização de procedimentos cirúrgicos é de grande importância nos dias atuais em função da dificuldade crescente de treinamentos in vivo. Assim, os recursos audiovisuais favorecem a maximização dos recursos vivos usados no ensino, e minimizam os problemas advindos somente com o verbalismo.

**Objetivo:**

Avaliar a utilização de um vídeo digital como estratégia pedagógica na disciplina de técnica operatória de um curso de medicina.

**Método:**

Estudo transversal com 48 acadêmicos do terceiro ano de medicina, cursando a disciplina de técnica operatória, divididos em dois grupos com 12 duplas, ambos submetidos ao método convencional de ensino, e um deles também exposto ao método alternativo (vídeo) demonstrando a técnica. Todos realizaram flebotomia no laboratório de experimentação, com avaliação do professor/monitor durante execução. Ao final, responderam a um questionário autoaplicável referente ao método de ensino e realização da técnica.

**Resultados:**

A maioria dos que não assistiu ao vídeo levou mais tempo na execução, fez mais questionamentos e necessitou de maior auxílio dos avaliadores. O total dos expostos ao vídeo seguiu a cronologia de execução e aprovou o novo método; 95,83% sentiram-se aptos para repetir o procedimento, e 62,5% daqueles submetidos apenas ao método convencional declararam ter capacidade regular de assimilação da técnica. Em ambos os grupos grande parte mencionou ter dificuldade regular, porém os que não viram o vídeo tiveram maior dificuldade na realização da técnica.

**Conclusão:**

O método tradicional de ensino associado ao vídeo favoreceu a capacidade de compreensão e transmitiu segurança, sobretudo por ser atividade que requer habilidade técnica. A visibilização da técnica motiva e desperta o interesse, facilita a compreensão e memorização dos passos para a execução do procedimento, beneficiando o desempenho dos acadêmicos.

## INTRODUCTION

The medical school requires academic students to understand many fundamental and highly complex concepts as well as it demands that they develop skills and abilities to carry out practical procedures. At the same time, the scientific and technological progress, the internet and the speed in which information and knowledge in the present context are generated, significantly influence the evolution and the activities in medicine.Therefore, the medical training demands for changes and improvements, which lead to changes in the teaching-learning process.

The National Academies of Science BIO 2010 Commission recommends the use of any appropriate technology to improve the students' understanding of their objects of study^5^ . Innovations in educational strategies can result in the evident progress in the practice of the contents learned, and also they may awaken greater interest from the students. As every educating process involves the relationship between learner and instructor, both participating and consciously interacting in the pursuit of a common goal, we highlight the important role of teachers whose performance requires the constant updating of the elements that are necessary for the teaching practice ^9.^

In medicine, such strategies should include the observation and active participation in practical activities, including surgical procedures, once such experiences contribute to the development of technical skills. The saying "practice makes perfect" makes believe that by training some technique to exhaustion will allow one master it. Therefore, the crucial point is: in order to achieve excellence one must first have full knowledge of what they want to accomplish, articulating the theoretical practical basis concerning the surgical technique, so that, by practicing it, one can train repeatedly until they reach quality and ability to perform a surgery.

The development of a means that may allow the observation of the procedure in these circumstances is of great importance because audiovisual resources favor the assimilation and minimize the problems that might are from the verbalism^4 ^. Similar methods have been used in other studies, in medicine and other areas, with good results. According to the authors, such methods favor learning, reduce the number of errors during practice, with a significant improvement in the performance of those who were exposed to videos as well as greater satisfaction and acceptance by the target audience 5 ^,^ 16 ^,^ 1 ^, 10 6,^ 7. It is assumed that the use of this visual resource can be beneficial for the understanding of the surgical technique, since it integrates the image of the teacher to the practical demonstration of the procedure, in great detail, illustrating and providing technical and scientific subsidies. Besides that, they can transmit confidence to the future doctors, when one is required to carry out such procedures throughout internships or in their actual works.

Given the above, this study aims to evaluate the use of a digital video as a pedagogical strategy in the surgical technique discipline from the course of medicine linked to the traditional method, and to assess their effectiveness in the teaching-learning process.

## METHODS

 It's a Cross-sectional study with a quantitative approach, approved by the Research Ethics Committee of the institution (No. 325 510). It was conducted in the discipline of Surgical Techniques from the Medical School of the Faculdade Evangélica do Paraná - FEPAR, Curitiba, Brazil. The targeted audience was composed by medical students, aged 18 or more, and who agreed to participate in the study by signing the informed consent form. The work was developed in five stages. In the first stage a video that displays the performance of the selected surgical technique was shown; the second consisted of a traditional lesson on that technique; in the third the students were exposed or not to the video; in the fourth they performed the procedure; and the last one was the evaluation of the teaching method to which students were submitted (a traditional classroom associated or not the digital video).The procedure was recorded and later burned to a DVD, by a specialized company. The main professor of the discipline participated in the recording. He also carried out the procedure with the help of monitors.

A total of 48 students from the third year of the Medical School of an institution participated in the study. 24 of them in 2013 and 24 in 2014, while attending the course. The participants were divided into two groups: group 1 (12 pairs) was submitted only to the usual teaching method (theoretical, practical and theoretical complement with the book); Group 2 (12 pairs), beyond the usual method, they were exposed to the alternative teaching method (audiovisual resource) twice: one time on the day before the class, and another time immediately prior to the first performance of the selected surgical procedure.

Two pairs of students performed the same technique in experimental animals and both members had or not, equally, access to the recording, however one of the pair did it on the right side of the neck and, then, on the left side. The setting of the pairs was organized by one of the lab's monitors who was also responsible for the technical presentation in digital video (DVD) and therefore the only one to know and record which pairs watched or not the audiovisual material.The professor and the second monitor, both in charge of assessing the students, were unaware of which pairs had access to the recording in order to maintain the impartiality of the evaluation.

**FIGURE 1:**
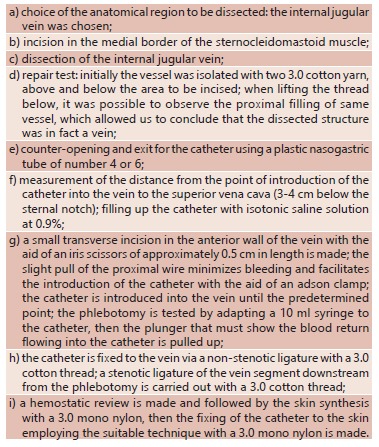
- Surgical steps for the execution of phlebotomy requested in the discipline of Surgical Technique from the Faculdade Evangélica do Paraná, Curitiba, PR, Brazil.

The technique chosen for the assessment of the method was phlebotomy in the cervical region and was carried out in the experimentation lab at FEPAR. This choice was based on two main reasons: it is relatively a fast technique and at the same time, requires from the student the skill and the knowledge of the steps (Figure 1) necessary for a successful performance. The animal chosen for the experiment was the Landrace pig, in a total of 12 animals, males and females, adults, healthy in their physical examination, weighing between 25 and 32 kilos, approximately four months old, which were donated by the FEPAR and from a local refrigerator. The animals were induced to anesthesia with tiopental and kept with isoflurane inhalation. When necessary it was used midazolam, ketamine, adrenaline and atropine. At the end, the animals were killed with thiopental intravenous (10 ml) followed by 19.1% of potassium chloride (20 ml).The entire process followed ethical norms directed to experimental animals.

 In the laboratory, during the practical classes of Surgical Technique, each pair of students was distributed among the animals at the practical lessons to perform the phlebotomy under the supervision and assessment of the professor and the second monitor. The setting of the pairs was of free choice among the students of the same group, and the sequence of the performance was drawn by the first monitor (which applied the video), thus avoiding a default order among those who watched or not the video.

For teaching and evaluation purposes, the technique was divided into three steps: 1) skin incision up to the repair of the internal jugular vein; 2) phlebotomy until the insertion and correct positioning of the catheter; 3) distal restraint to fixing the catheter through the appropriate technique, hemostasis and skin suturing.Thus, the professor and the second monitor assessed whether the students followed the recommended chronological order of the steps for the technical execution. Also, they assessed the total time of the performance, the fragmented time according to each step, and the number of times the professor was questioned throughout each stage and if there was any interference during the practice by the professor or the monitor.

**FIGURE 2:**
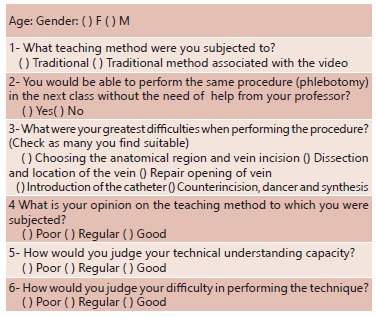
- Questionnaire answered by students

After the procedure, the students individually answered a self-administered questionnaire with multiple questions, which addressed issues related to the teaching method and the realization of the surgical technique (Figure 2). The method of data analysis used to evaluate the responses was the chi-square test, using percentages to present the results of each group. Then, in the assessment of the students as for their practical performance of the procedure the time, the number of questions and interventions of the professor / monitor were pondered, by applying the "t" test student for the analysis of these variables. For both evaluations it was adopted a significance level of 5%.

## RESULTS

Out of the 48 participants in the study, 25 were men and 23 women, aged between 19 and 28, and an average of 21.6 years. It is worth mentioning that only those who, at time of the research, had no previous training in surgery, were selected.

The results from the practical assessment (Table 1) show the time used by each pair of students to perform the procedure, the number of questions they had, and the number of interventions by the professor and / or monitor while performing the surgical technique. 

**TABLE 1:** - Practical evaluation on the implementation of surgical technique

	**Execution time (in minutes)**	**Number of questions (per pair)**	**Number of interventions (by professor or monitor)**			
Students	Group 1	Group 2	Group 1	Group 2	Group 1	Group 2
Video Animal	No	Yes	No	Yes	No	Yes
Pig 1	59:00	46:00	2	4	4	4
Pig 2	48:58	32:57	14	7	7	2
Pig 3	40:00	20:00	40	1	19	0
Pig 4	47:00	67:12	4	8	2	6
Pig 5	42:17	64:35	33	28	2	1
Pig 6	48:00	31:00	6	1	4	3
Pig 7	70:00	36:06	23	4	16	0
Pig 8	46:26	35:08	10	6	12	1
Pig 9	46:11	42:15	10	5	8	2
Pig 10	51:23	50:58	8	3	7	0
Pig 11	66:46	64:02	35	10	19	3
Pig 12	47:00	26:00	11	2	9	0
Total	633: 04	516: 13	196	79	109	22
Average	51:05	43:01	16.34	6.58	9.08	1.83
Median	47:30	39:10	10.5	4.5	7.5	1.5
Standard deviation	0.389	0.657	13.068	7,292	6,141	1,898
P- value	0.1176		0.0181		0.0051	

Group 1 = students exposed to the conventional technique; Group 2 = students exposed to video and conventional technique.

Out of the 24 pairs of academic students, 12 in 2013 (pig 1-6) and 12 in 2014 (pig 7-12), the majority - 10 out of 12 pairs (83%) - who were submitted to the usual method of teaching associated to the alternative one (Digital video) performed the procedure in less time than the one used by the pairs who used only the traditional teaching approach. The time difference (Table 1) between the pairs who watched the video compared to the timing of the ones who didn't was not significant (p=0.1176), averaging 43:01 minutes for the group who was exposed to the video and 51:05 min for the group who wasn't. The pair with the longest execution time (70:03 min) did not watch the video and the pair with the shortest time (20:00 min) are among those who did see the explanatory video.

Table 1 also illustrates the number of questions from each group, with a total of 196 questions in group 1 (did not watch the video) averaging 16.34 questionings, and 79 from group 2 (watched the video) with an average of 6.58. The difference between the two groups was significant (p=0.0181).

As a result, 100% of the students exposed to the new method (group 2) complied with the chronological order, requiring the intervention of the professor in an average of 1.83 times per pair. On group 1, five of the 12 pairs (41.6%) did not follow the chronological order for the execution of the procedure, averaging 9.08 of interventions by the evaluator in order for the procedure to continue. The described level was significant (p=0.0051).

The analysis of students' responses, referring to the questionnaire, shows that among the 12 pairs subjected to the traditional method of teaching associated to visual aid (group 2), 95.83% reported feeling able to perform the procedure again without any help from the professor (question 2 of questionnaire). By contrast, 75% of students exposed only to the usual teaching method (group 1) said the same (p=0.04897).

**FIGURE 3:**
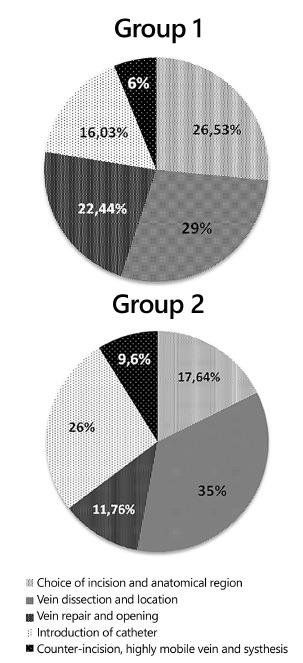
- Difficulties pointed by academic students in performing the procedure

Both groups reported having some difficulties during the execution of the surgical technique (question 3), with a higher number of difficulties (49) appearing in group 1 (traditional method) in comparison to group 2 (34 difficulties). In both groups, the biggest problem occurred during the dissection and location of the vein, 29% of such in group 1 and 35% in group 2. In relation to other surgical steps of the technique, the group which was not exposed to the video had more difficulty in choosing the anatomical region for the incision (26.53%) and the execution of the repair test (22.44%), whereas group 2 presented 17.64% and 11.76% of difficulty, respectively (Figure 3). Group 2 had as second highest difficulty (26%) the introduction of the catheter (p=0.4953).

When asked (question 4) on the exerted teaching strategy, 100% of students who watched the video considered the association of this feature to the traditional a good teaching method, whereas those among whom did not watch the video, 62.5% indicated the method as fair, 25% good and 12.5% ​?as bad ​as bad (p=0,00000055739).

Regarding the technical understanding capacity (question 5), the majority (83.34%) of the students who were exposed to the video evaluated having good assimilative capacity of the procedure and 16.66% considered their ability as regulate, while 62.5 % of those subject only to the conventional method claimed to have regular capacity for understanding the technique, 33.34% pondered it as good and 4.16% as bad (p=0.0019).

Great part of the students who did not watch the video deemed having regular difficulty (83%) in the execution of the technique (question 6), whereas the minority considered their difficulty as good (9%) and as bad (8%). Among those who watched the video, the majority also claimed to encounter regular difficulty (75%) in the execution and 25% rated it as good. None of the students exposed to the video thought they had bad difficulty (p=0.1283).

## DISCUSSION

The study was based on two different forms of evaluation between the groups. The first expounded in the evaluation of the direct practice of students by measuring the time used in the procedure, amount of inquiries carried out and the number of necessary interventions made ​​by the professor/monitor. The second consisted of the application of a questionnaire, which estimated the students' performance considering the method to which they were exposed, analyzing difficulty and safety of the technique from the point of view of the students themselves.

It is believed that previous experiences in the surgical field that enable the development of specific skills, volunteer placements, the intrinsic ability of each to cope with handling tools, and even the stress and responsibility of a surgical procedure, are as influential as a good teaching strategy, i.e. they represent realities that neither lectures nor videos can provide. However, not only ability can and should be taken into account.

In analyzing the study profile of the doctor throughout their academic and professional life, a survey found that the medical student prioritize theoretical models and abstract concepts subjects, while resident doctors and experts recommend the practicing of the concept as a form of learning. According to the authors, this emerges as a limitation for learning, as when faced with the practical exercise of theory, students realize that not everything is as expected, as planned^2^. In function of what has been observed, one sees the need to develop and implement new teaching alternatives.

The practical assessment in this study demonstrated that the performance of the undergraduates who were submitted to the conventional method of teaching associated with exposure to audiovisual resource, in general, was better than those exposed only to the usual method. Although the time difference for the execution of the procedure was not statistically significant between groups, the analysis of descriptive data exposure indicates that the audiovisual method was relevant to the learning of the students, for those who had the opportunity to use it had better performance when executing the surgical technique, both with respect to time and in number of inquiries and need for interventions by the teacher and / or monitor.

This reinforces the fact that prior visualization of the procedure shown in video favored understanding and memorizing the steps to be followed by undergraduates to reproduce the technique of phlebotomy, as group 2 had less doubts and required less interference from the professor. It is not surprising that both groups presented more difficulty at the time of the location and dissection of the vein, since this step requires more skill and experience, which is achieved by the repetition of the activity. It is worth pointing out that, according to the evaluators, the difficulty encountered by some pairs in the group that was exposed to video in dissection and location of the vein, and the introduction of the catheter, is due to the anatomical conditions of the animal, and that this group showed less uncertainty during the execution of the procedure.

A study on the instructional video production process and its use as a teaching strategy highlights that when well planned and with adequate audiovisual language, its insertion displays the image of the teacher explaining and demonstrating content "in great detail and information" which favors the understanding of the undergraduate; However, the authors caution that this feature should not be used alone 12. In other words, the video allows students to mentally construct the whole process they must follow more effectively^15^, without excluding the teacher, whose role consists of planning and enhancing the application of this feature in order to spark interest, improve understanding and unleash the critical and reflective thinking.

The answers in the questionnaire of the group who watched the educational video shows that these felt more confident about the technique and fully satisfied with the teaching method presented, as opposed to the other group. Such facts are fitting to the results of a bibliographical review on the application of surgical simulation models and educational videos used in surgical training, whose survey showed that both videos and surgical techniques simulators favor the learning and development of skills for performing procedures. 

These results are congruent with those presented in other researches that sought to combine different teaching methods, including technology, to traditional teaching^5, 7, 15, 8, 3,^ and highlight the importance of pre visualization of conduct, regardless of the mean. However, none of the works are able to suggest that the same applies to highly complex procedures, as they sometimes require more than just theoretical and practical knowledge of the procedure. They also require experience.

Areas such as anatomy and surgery, which are very visual, perfectly adapt to these innovations because they require numerous details and spatial understanding of the situation^15^. The advantage is that the cost to implement these new methods becomes relatively low over time because, from the start, the production of material can be expensive, but a single format is applicable to hundreds of students, and indefinitely 4 ^, 15.^

While acknowledging the effectiveness of the new features to expand the sources of information, one cannot neglect the classical method because it is the main foundation able to guide and awaken the interest of the student. With the teacher as the primary factor in learning, able to define personality and show the paths to knowledge, it is important to be capable of recognizing new teaching methods, and then integrate them into already used ones, to enable the student to be more active in the process of building their knowledge^14^. The acquirement of methods in conjunction is superior to any that acts singularly^1^ ^, 14.^

New technologies may have educational significance; however, in order to realize the actual effect , it is necessary for the staff to be able to deploy the new method, and, simultaneously, for the material to have good quality^11^^,^ i.e. the implementation of any new developments in the academic environment can not occur randomly^15^. However, some authors suggest the of any special education resource that allows the student to have greater access to information and will facilitate the best theoretical and practical understanding of a subject 15 ^, 11.^

We should understand by all and any recourse, those who indeed "contribute to learning" and "represent added value to the work of the professor," further qualifying the activity of teachers in the education process of future professionals^13^.

Although some variables were not evaluated in this study - that is, the intentions and individual aptitude of undergraduates for surgery, didactic and anatomical conditions of the animals, as well as the educational quality and aesthetic characteristics of the video - the study showed that the use of this technology as a teaching strategy can motivate the participation of academic students and influence their ability to learn, especially in those activities that require technical skills. 

Another aspect to consider is the option for phlebotomy. It is suggested to replicate the study with other procedures in order to highlight the contribution and effectiveness of this form of visual aid. It is also clear that the usual method of teaching is essential, considering the theoretical and scientific foundations, but becomes more efficient when combined with didactic and pedagogical alternatives, especially those that add the visualization of what you want to teach. 

The fact that most of those who watched the video, apparently, showed more confidence to perform the procedure, reflects their approval as for the association of methods and that it did facilitate the understanding of the technique.

From another perspective, it is worth warning that there is little research on the production of audiovisual materials with respect to the necessary materials for the teaching-learning process, in other words, to guide the development of adequate educational materials, one that are able to complement and integrate the theory to the challenge to implement the act in practice, which in the case of this study, it is a surgical procedure. 

## CONCLUSION

The traditional method of teaching associated with video favored the ability to understand and transmitted safety, particularly because it is an activity that requires technical skill. The visualization of the technique motivates and arouses interest, and facilitates the understanding and memorizing of the steps for executing the procedure, thus benefiting the performance of academics.
